# A Novel Prophylactic Device Against Head Deformity to Prevent Severe Positional Plagiocephaly

**DOI:** 10.3390/jcm14093261

**Published:** 2025-05-07

**Authors:** Yukari Tanaka, Hiroshi Miyabayashi, Takanori Noto, Risa Kato, Nobuhiko Nagano, Ichiro Morioka

**Affiliations:** 1 Department of Pediatrics and Child Health, Nihon University School of Medicine, 30-1, Oyaguchi, Kami-cho, Itabashi-ku 173-8610, Japan; tanaka.yukari538@nihon-u.ac.jp (Y.T.); noto.takanori@nihon-u.ac.jp (T.N.); kato.risa@nihon-u.ac.jp (R.K.); 2 Department of Pediatrics, Kasukabe Medical Center, 6-7-1, Chuo, Kasukabe-shi 344-8588, Japan; miyabayashi@dr.memail.jp

**Keywords:** cranial shape, device, positional plagiocephaly, prevention

## Abstract

**Background/Objectives**: Preventing head deformity in the early postnatal period could avert positional plagiocephaly (PP). Accordingly, we developed a novel prophylactic device to prevent head deformity and examined its impact on the incidence of PP and prevention of severe PP at 3 months of age. **Methods**: The newly developed prophylactic device was used immediately after birth or at the 1-month checkup, and cranial shape was measured before device application and at 3 months of age. The diagnostic threshold for PP was >5% for cranial vault asymmetry index (CVAI); cranial asymmetry (CA) of ≥13 mm was deemed severe. A database comprising cranial geometry of 3-month-old healthy Japanese infants (*n* = 110) served as the control. **Results**: This study included 42 infants who started using the novel prophylactic device immediately after birth or at the 1-month checkup. Measurements at 3 months of age revealed that the prophylactic device group had significantly lower CA and CVAI than the control group (CA [median]: 5.5 vs. 8.0, respectively, *p* = 0.007; CVAI: 4.3 vs. 5.8, respectively, *p* = 0.048). However, the PP prevalence did not differ significantly between the two groups (41% vs. 57%, respectively, *p* = 0.094). The number of infants with severe PP was significantly lower in the prophylactic device group than in the control group (0% vs. 14%, respectively; *p* = 0.012). At 3 months of age, no significant differences in CA or CVAI were observed between the immediate postnatal and 1-month groups. **Conclusions**: The novel prophylactic device against head deformity could prevent severe PP.

## 1. Introduction

The skull of newborns and infants is soft, and prolonged external pressure on the same part of the skull due to orientation can easily lead to skull deformation, resulting in positional plagiocephaly (PP) [1,2,3]. In 1992, the American Academy of Pediatrics recommended that infants should not be placed in a prone position during sleep to prevent sudden infant death syndrome (SIDS). Although the incidence of SIDS decreased, that of PP, which was estimated to be 0.3% before the recommendation, increased to 48% [4,5]. The spontaneous incidence of PP reportedly increases from 19% after birth to 50% and 56.8% during the first and third months of life, respectively, and then decreases to 43.2% at 6 months of age when babies exhibit full head control [6,7].

Preventive methods for PP include physiotherapy [8], including postural change and tummy time; however, in cases of severe PP, physiotherapy alone has limited preventive effects, and helmet therapy is employed [9]. However, helmet therapy is not covered by the Japanese health insurance, resulting in a considerable financial burden. Furthermore, helmet therapy is not widely accepted because its long-term use may cause pressure ulcers, dermatitis, or other adverse events [10]. Moreover, a systematic review of 19 studies in 2017 suggested that head deformity may affect infant growth [11]. Preventing head deformity is important not only from a cosmetic perspective but also for good infant growth and development.

We hypothesized that head deformity could be prevented by prophylactic measures implemented immediately after birth, given that head deformity is uncommon immediately after birth. In November 2022, the American Academy of Pediatrics recommended that soft positioning pillows should not be used to prevent head deformity [12]. Therefore, we aimed to develop a safe prophylactic device to prevent head deformity and successfully developed a device against head deformity (Japanese patent application number: 2023-025276).

In this current study, we aimed to determine whether the onset and progression of PP can be safely prevented at 3 months of age in infants who begin using the developed prophylactic device against head deformity by 1 month of age.

## 2. Subjects and Methods

### 2.1. Control Cases

The participants were full-term infants whose parents were Japanese and who were born at Nihon University Itabashi Hospital and Kasukabe City Medical Center during a 10-month period between 1 March and December 2023. The participants were infants whose parents had given informed consent immediately after birth or a 1-month health check. All parents who could be eligible were made aware of the study and given the opportunity to apply during the study period. Infants with a history of neonatal asphyxia with an Apgar score ≤ 7 at 5 min or cephalhematoma were excluded. In addition, two different neonatologists examined infants within 48 h of birth or 1-month check up to exclude those with congenital abnormalities of cranial shape (e.g., craniosynostosis).

### 2.2. Novel Prophylactic Device Against Head Deformity

We developed a novel prophylactic device against head deformity in collaboration with Berry Inc. (Japanese patent application number: 2023-025276; Figure 1). The device is designed to prevent head deformity by making the surface adjacent to the skull coincide with the cranial shape. The device comprises three layers: a resin shell that prevents cranial flattening; a cushion that prevents skin problems; and a cover. The shell size corresponds to +2 standard deviation of the Japanese head circumference at 3 months of age; thus, it can be used continuously from birth up to 3 months of age. It is also designed to prevent forward tilt, thus avoiding suffocation.

The participants were loaned the novel prophylactic device against head deformity and instructed to place the device on the back of the head throughout the period, from immediately after birth or 1 month after birth to 3 months of age, while lying in the supine position.

### 2.3. Cranial Shape Measurement

**Figure 2:**
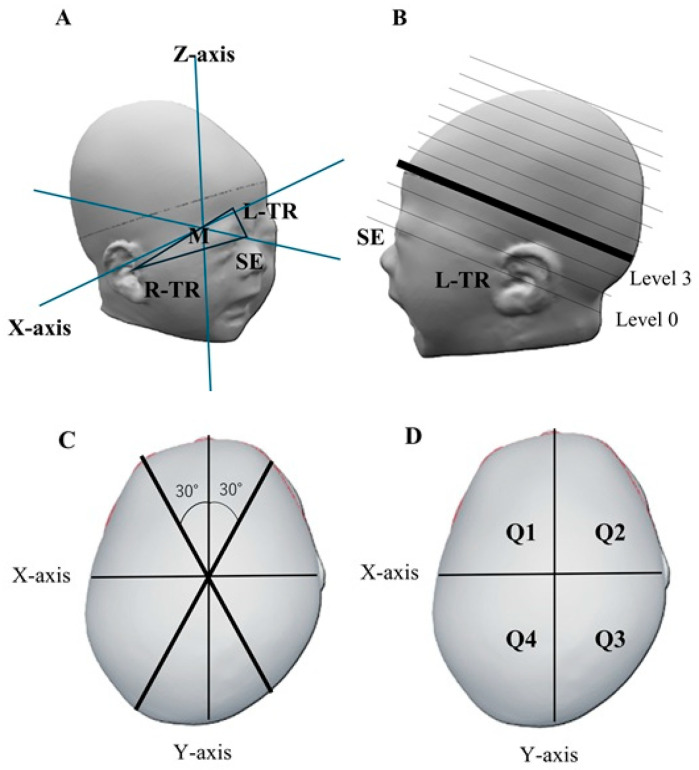
Method of measuring cranial shape. (**A**) Method of determining the X-axis, Y-axis, and reference plane. The plane connecting the three points SE, L-TR, and R-TR is defined as the reference plane (level 0). The line connecting the midpoints M and SE of both TRs is the Y-axis. On Level 0, the X-axis is a line passing through M and perpendicular to the y-axis. (**B**) How to determine the Z-axis and each level. (**C**) Diagram of the measurement surface (level 3). Measure the distance of a straight line at 30° from the y-axis. (**D**) How to measure the volume ratio. Divide the head into 4 parts on the X-axis and Y-axis planes. Q 1: Left frontal head, Q2: Right frontal head, Q3: Right occipital head, Q4: Left occipital head, L-TR: Left tragion, M: Midpoint of both TRs, R-TR: Right tragion, SE: sellion, TR: tragion.

The cranial shape was measured using a 3-dimensional stereophotogrammetry (VECTRA^®^H2; Canfield Scientific, Parsippany-Troy Hills, NJ, USA) at two distinct time points: before using the prophylactic device against head deformity and at 3 months of age. The plane connecting three points, namely the sellion (the lowest point of the nasal root; SE) and the right and left tragions (the upper margin of the tragus; TR), was defined as the basic plane (level 0). The midpoint M of the two TRs was set, and a line passing through SE and M was defined as the Y-axis; the X-axis was a line perpendicular to the Y-axis passing through M on the plane of level 0 [13,14] (Figure 2A). Ten equal cross-sections of the cranium above level 0 were constructed. Each cross-section or level was determined by dividing the height from the top of the level 0 plane to the apex of the head into 10 equal sections [14,15]. The level 3 cross-section was used as the measurement plane [14,15,16]. Figure 2C,D shows a cross-sectional view of the measurement plane. Cranial length (CrL) and cranial width (CrW) were measured as the length of the measurement plane in the Y- and X-axis directions, respectively.

### 2.4. Head Deformity Parameters

This present study evaluated the cephalic index (CI), cranial asymmetry (CA), cranial vault asymmetry index (CVAI), anterior symmetry rate (ASR), and posterior symmetry rate (PSR) as symmetry-related parameters [17,18,19,20]. CI was calculated as CrW/CrL (%) [16]. CA was defined as the difference between the longer and shorter axial lengths of 30° from the Y-axis of the measurement plane to the left and right, respectively (Figure 2C). Furthermore, CVAI was defined as CA/shorter axial length (%) [18,21].

The four regions of the head, divided by the x- and y-axis planes, were defined as left frontal (Q1), right frontal (Q2), right occipital (Q3), and left occipital (Q4) regions, respectively (Figure 2D) [20]. ASR was defined as the ratio of the smaller to the larger volume in Q1 and Q2 (Q1/Q2 or Q2/Q1; the denominator is larger). Likewise, PSR was defined as the occipital volume ratio [14,20].

Among Japanese individuals, the diagnostic threshold was a CVAI value of >5% [6,22,23], and a CA value of ≥13 mm was defined as severe [23].

### 2.5. Items for Consideration

The following questions were examined to determine whether using the novel prophylactic device against head deformity could affect cranial shape at 3 months of age. Safety was also evaluated.

(1) Can the use of the present novel prophylactic device prevent head deformity at 3 months of age?

Cranial shape parameters in infants who used the device by 1 month of age were compared with previously reported data from healthy 3-month-old infants [24]. We also compared the prevalence of PP and the incidence of severe PP.

(2) Is there a difference in cranial shape at the start of device use and at 3 months of age?

We determined whether cranial shape parameters in infants at 3 months of age differed between the infant group that started using the device immediately after birth and the group that initiated device use at 1 month of age.

### 2.6. Statistical Analysis

For each parameter, the Shapiro–Wilk test was used to determine the distribution, and the median and interquartile range were used for non-normally distributed parameters. The Mann–Whitney U-test was used for intergroup comparison. The chi-square test was used to test frequencies. A two-tailed test with *p* < 0.05 was considered statistically significant. EZR (ver 1.61) was used for statistical processing [25].

## 3. Results

### 3.1. Patient Background

Of the 53 infants who started using the device immediately after birth during the study period, 21 dropped out by measurement at 3 months of age, and 32 continued using it for 3 months (immediate postnatal group). None of the participants for whom consent was obtained met the exclusion criteria. Additionally, 10 infants started using the device at 1 month of age and used the device until 3 months of age (the 1-month group). Forty-two patients who used the device until 3 months of age were examined. Table 1 summarizes the clinical backgrounds of all infants, as well as those categorized by group. There were no differences in the clinical backgrounds between the immediate postnatal and 1-month groups.

### 3.2. Comparative Assessment of Cranial Shape at 3 Months of Age

Table 2 presents the changes in cranial shape parameters in 42 infants before and at 3 months after starting prophylactic device use. Table 3 shows the sex ratio, age at the time of measurement, cranial shape parameters, and prevalence of PP compared with the control group (*n* = 110 infants). At 3 months of age, infants in the prophylactic device group had significantly fewer deformities in CA, CVAI, and PSR than those in the control group. However, no significant difference in the prevalence of PP was observed. A chi-square test was performed on the number of infants with severe PP by dividing those with CA ≥ 13 mm and those with CA < 13 mm into two groups. The prophylactic device group had significantly fewer cases of severe PP than the control group (0 vs.15, respectively; *p* = 0.012).

### 3.3. Timing of Device Initiation

In the current study, the prophylactic device use was started immediately after birth or at the 1-month checkup. Table 4 presents the sex ratio, age at the time of measurement, and cranial shape parameters for determining differences between the timing of device use and the preventive effect against PP. There were no significant differences in any of the cranial shape parameters.

### 3.4. Device Safety

In total, 63 infants used the newly developed prophylactic device and experienced no adverse events such as suffocation (including attempted suffocation) or dermatitis during the study period.

## 4. Discussion

In this study, we determined whether our novel prophylactic device facilitates the prevention of cranial deformity up to 3 months of age. We found that our novel prophylactic device could safely prevent the progression of cranial deformity.

### 4.1. Safety of the Prophylactic Device Against Head Deformity

The risk of SIDS is a major factor, as the American Academy of Pediatrics currently recommends that positional pillows should not be used. Although soft bedding such as pillows can carry a risk for SIDS [26], the prophylactic device against head deformity used in this study was designed to prevent forward tilt and lacked materials capable of causing suffocation. The device was considered safer than soft pillows sold in the general market. During the study period, no risk events related to suffocation were reported in association with the device use. Additionally, cushions and covers other than the hard shell can be washed and replaced, and users did not experience any skin-related issues.

### 4.2. Methods of Measuring Cranial Geometry

Stereophotogrammetry was employed in this work to create the 3D data files. Stereophotogrammetry can generate 3D data for analyzing an infant’s cranium without radiation exposure or specialized training. This device can measure cranial geometry without radiation exposure, but computed tomography and magnetic resonance imaging have been used to determine cranial geometry, and pediatric radiology consultation is also important when necessary [27]. Methods that do not use ionizing radiation, such as gradient echo black-bone and zero time echo MRI sequences, are at the research stage and are the subject of future studies [28].

### 4.3. Prevention of Head Deformity

Significantly fewer head deformity parameters were detected in the prophylactic device group than in the control group. Moreover, initiating prophylactic device use immediately or 1 month after birth could significantly reduce head deformity at 3 months of age. Additionally, the incidence of head deformity at 3 months of age did not differ significantly between the immediate postnatal and 1-month groups, indicating that head deformity at 3 months of age can be prevented regardless of the timing of initiation of prophylactic device use.

### 4.4. Prevention of Severe PP

We examined the preventive effect of the novel prophylactic device against severe PP, revealing that infants in the prophylactic device group did not develop severe PP. A statistically significant difference was observed between the prophylactic device and control groups, indicating the preventive effects of the prophylactic device on the onset of PP and progression to severe PP.

### 4.5. Benefits of Preventing Head Deformity

Head deformity may be a risk factor for developmental delay [11], indicating that prevention of head deformity may be beneficial from the perspective of infant growth. Preventing head deformity could circumvent the need for therapeutic intervention, thereby rendering the financial burden of introducing helmet therapy unnecessary. Moreover, no skin problems, such as pressure ulcers or dermatitis, occurred during the use of the novel device examined in this study, resolving the disadvantage of skin problems [10] frequently associated with helmet therapy.

### 4.6. Future Lines of Research

This study could be an important turning point in creating a paradigm shift from conventional treatment of cranial deformities in infants to prevention of cranial deformities in infants. The future plan is to establish a method to eliminate cranial deformities in the Japanese population through a large-scale intervention that includes both education of parents on cranial shape prevention and the use of the device.

### 4.7. Limitations

This study has limitations in terms of sample size and duration of device use. The participants were limited to only two facilities in Japan, which hampered the generalization of our findings. Nevertheless, the use of the novel prophylactic device against head deformity enables the safe prevention of severe PP, which is of clinical significance. The efficacy of the present device needs to be confirmed in future prospective clinical studies using a larger sample size. In this study, we could not determine the specific duration of device use. Additionally, there were individual variations; specifically, infants’ movement increased with increasing age in months, thereby reducing the duration of device use owing to device misalignment. Furthermore, the actual time of fitting was also inadequately described. Further improvements in the device are needed to prevent the misalignment of the device and extend the usage time as much as possible. In this present study, it was not possible to investigate the association between child nutrition and cranial shape. The relationship between breastfeeding and cranial deformity was considered an issue for future study.

## 5. Conclusions

In this current study, we examined the use of our novel prophylactic device against head deformity to prevent the onset and progression of PP. Our findings suggest that the use of the present novel device could safely prevent the onset and progression of PP.

## 6. Patents

A patent for the novel prophylactic device against head deformity was filed with the Japanese Patent Office on 21 February 2023, by Mitsuhiro Sakata, Yuutaro Nakoshi, Nobuhiko Nagano, Risa Kato, Ichiro Morioka, and Hiroshi Miyabayashi (Application number: 2023-025276).

## Figures and Tables

**Figure 1:**
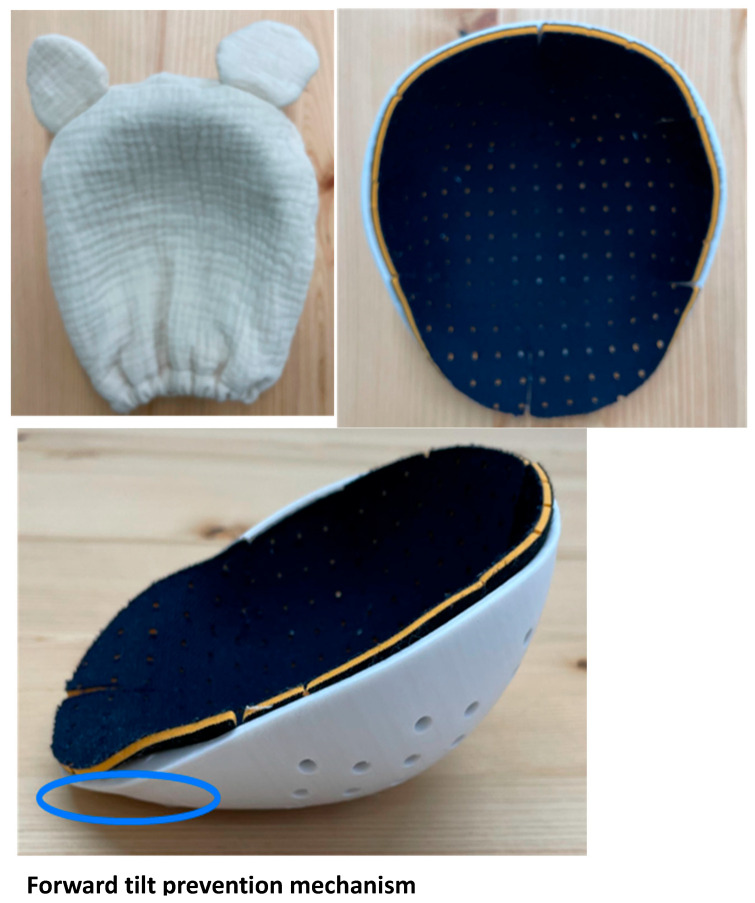
Novel prophylactic device against head deformity. The cranial contact surface follows the shape of the cranium to prevent cranial deformation beyond the product shape. The product consists of three layers: a resin shell that prevents cranial flattening; a cushion to prevent skin problems; and a cover. The shell has a head circumference of +2 standard deviation at 3 months and can be used continuously from birth up to 3 months of age. The shell is also designed to prevent tilting forward, thereby preventing suffocation.

**Table 1:** Comparison of infant backgrounds according to the timing of initiation of the novel prophylactic device against head deformity.

	Total (*n* = 42)	At Birth (*n* = 32)	At 1 Month of Age (*n* = 10)	*p*-Value
Male:Female	15:27	10:22	5:5	0.280
Number of deliveries (primiparous, multiparous)	18:14	22:10	6:4	0.608
Delivery method (vaginal, cesarean)	27:15	18:14	9:1	0.052
Gestational weeks	39 (±1.3)	39 (±1.3)	39 (±1.2)	0.604
Birth weight (g)	2947 (±377)	2947 (±392)	2946 (±323)	0.998
Head circumference (cm) at birth	33.2 (±1.1)	33.2 (±1.1)	33.2 (±1.4)	0.791

Data are presented as the number of infants or mean ± standard deviation.

**Table 2:** Comparison of cranial shape parameters before initiating the novel prophylactic device against head deformity and at 3 months of age.

(*n* = 42)	Before Device Use	At 3 Months of Age
CI (%)	84.4 [82.3–86.9]	88.9 [84.4–93.5]
CA (mm)	2.8 [1.2–5.1]	5.5 [2.3–9.1]
CVAI (%)	2.3 [1.1–4.3]	4.3 [1.8–7.2]
ASR (%)	95.4 [91.3–98.6]	95.7 [91.7–97.7]
PSR (%)	93.8 [87.7–96.6]	92.4 [88.8–96.5]
Number of infants with positional plagiocephaly (%)	9 (21)	17 (41)

Data are presented as median (interquartile range) or the number of infants (percentage). ASR, anterior symmetry rate; CA, cranial asymmetry; CI, cephalic index; CVAI, cranial vault asymmetry index; PSR, posterior symmetry rate.

**Table 3:** Comparison of cranial shape between the prophylactic device and control groups.

	Control Group (*n* = 110)	Prophylactic Device Group (*n* = 42)	*p*-Value
Male:Female	57:53	15:27	0.075
Age in days	100 ± 10.4	96 ± 6.3	0.019
CI (%)	88.7 [84.6–92.9]	88.9 [84.4–93.5]	0.670
CA (mm)	8.0 [4.4–11.3]	5.5 [2.3–9.1]	0.007
CVAI (%)	5.8 [3.2–8.1]	4.3 [1.8–7.2]	0.048
ASR (%)	94.7 [90.9–97.1]	95.7 [91.7–97.7]	0.210
PSR (%)	89.7 [83.9–94.4]	92.4 [88.8–96.5]	0.035
Number of infants with positional plagiocephaly (%)	63 (57)	17 (41)	0.094
Number of infants with severe positional plagiocephaly (%)	15 (14)	0 (0)	0.012

Data are presented as the number of infants, mean ± standard deviation, median (interquartile range), or the number of infants (%). ASR, anterior symmetry rate; CA, cranial asymmetry; CI, cephalic index; CVAI, cranial vault asymmetry index; PSR, posterior symmetry rate.

**Table 4:** Comparison of cranial shape parameters according to the timing of initiating the novel prophylactic device against head deformity.

	Started at Birth (*n* = 32)	Started at 1 Month of Age (*n* = 10)	*p*-Value
Male:Female	10:22	5:5	0.280
Age in days	95 ± 6.3	97 ± 6.5	0.441
CI (%)	89.4 [84.6–94.2]	88.3 [84.4–89.0]	0.170
CA (mm)	5.9 [3.7–9.2]	2.7 [2.0–6.2]	0.226
CVAI (%)	4.7 [2.8–7.2]	2.0 [1.6–4.8]	0.165
ASR (%)	95.5 [91.4–97.7]	95.9 [92.7–96.8]	0.859
PSR (%)	91.4 [86.8–96.1]	94.1 [89.7–99.1]	0.092

Data are presented as the number of infants, mean ± standard deviation, or median (interquartile range). ASR, anterior symmetry rate; CA, cranial asymmetry; CI, cephalic index; CVAI, cranial vault asymmetry index; PSR, posterior symmetry rate.

## Data Availability

The data presented in this study are available on reasonable request from the corresponding author.

## Abbreviations

ASR	Anterior symmetry rate
CA	Cranial asymmetry
CI	Cephalic index
CrL	Cranial length
CrW	Cranial width
CVAI	Cranial vault asymmetry index
PP	Positional plagiocephaly
PSR	Posterior symmetry rate
SIDS	Sudden infant death syndrome
